# Do choosing wisely recommendations about low-value care target income-generating treatments provided by members? A content analysis of 1293 recommendations

**DOI:** 10.1186/s12913-019-4576-1

**Published:** 2019-11-11

**Authors:** Joshua R. Zadro, John Farey, Ian A. Harris, Christopher G. Maher

**Affiliations:** 1 Faculty of Medicine and Health, Sydney School of Public Health, The University of Sydney, Sydney, NSW Australia; 2 Institute for Musculoskeletal Health, Sydney Local Health District, Sydney, NSW Australia; 3 Department of Orthopaedic Surgery, Royal Prince Alfred Hospital, Sydney, NSW Australia; 4 Ingham Institute for Applied Medical Research, South Western Sydney Clinical School, University of New South Wales, Sydney, NSW Australia

**Keywords:** Choosing wisely, Low-value care, Medicine, Surgery, Diagnostic, Allied health, Test, Treatment

## Abstract

**Background:**

It is unknown to what extent Choosing Wisely recommendations about income-generating treatments apply to members of the society generating the recommendations.

The primary aim of this study is to determine the proportion of Choosing Wisely recommendations targeting income-generating treatments, and whether recommendations from professional societies on income-generating treatments are more likely to target members or non-members. The secondary aim is to determine the prevalence of qualified statements, and whether qualified statements are more likely to appear in recommendations targeting income-generating or non-income-generating treatments that apply to members.

**Methods:**

We performed a content analysis of all Choosing Wisely recommendations, with data extracted from Choosing Wisely websites. Two researchers coded recommendations as test or treatment-based, for or against a procedure, containing qualified statements, income-generating and applying to members. Disagreements were resolved by discussion or consultation with a third researcher. A Chi-squared test evaluated whether society recommendations on income-generating treatments were more likely to target members or non-members; and whether qualified statements were more likely to appear in recommendations targeting income-generating or non-income-generating treatments that apply to members.

**Results:**

We found 1293 Choosing Wisely recommendations (48.3% tests and 48.6% treatments). Ninety-eight treatment recommendations targeted income-generating treatments (17.8%), and recommendations on income-generating treatments were less likely to target members compared to non-members (15.6% vs. 40.4%, *p* < 0.001). Nearly half of all recommendations were qualified (41.9%), with a similar proportion of recommendations targeting income-generating and non-income-generating treatments that apply to members containing qualified statements (49.4% vs. 42.0%, *p* = 0.23).

**Conclusions:**

Many societies provide Choosing Wisely recommendations that minimise impact on their own members. Only 20% of treatment recommendations target income-generating treatments, and of these recommendations mostly target non-members. Many recommendations are also qualified. Increasing the number of recommendations from societies that are unqualified and target member clinicians responsible for de-implementation of low-value and costly treatments should be a priority.

## Background

Choosing Wisely is a major public awareness campaign that aims to reduce low-value care through increasing discussions between patients and clinicians about the inappropriate use of medical tests and treatments [1]. Low-value care is care that provides little-to-no benefit or causes harm, provides a benefit too small given its cost, and is unlikely be desired by an adequately informed patient [2]. Choosing Wisely began in April 2012 as an initiative of the American Board of Internal Medicine (ABIM) Foundation, drawing inspiration from earlier initiatives, such as “Medicine’s Ethical Responsibility for Health Care Reform – The Top Five List” [3]. Today, Choosing Wisely is a global campaign with active campaigns in more than 15 countries (e.g. United States, Canada, United Kingdom, Australia, Italy, Netherlands). Choosing Wisely continues to expand and inspire the development of similar initiatives, such as EVOLVE [4] and the Value Based Insurance Design (VBID) Health’s Task Force on Low-value Care [5].

In an effort to reduce low-value care, professional societies from a variety of health disciplines (including surgery, medicine, diagnostics and allied health) have published Choosing Wisely lists [1]. Choosing Wisely lists comprise recommendations intended to facilitate open patient-clinician discussions about tests and treatments that are unnecessary, and that both patients and clinicians should question. There are over 1000 Choosing Wisely recommendations published worldwide but there is yet to be an evaluation of their content.

An ongoing concern is whether the recommendations made by professional societies focus on the low value care specifically provided by their members [6, 7]. Because of a lack of research it is unclear if recommendations typically refer to treatments provided by members of the society generating the recommendations, and whether recommendations are less likely to target treatments provided by members when income is involved. Ensuring recommendations refer to treatments provided by members (hereafter referred to as recommendations that ‘apply to members’) is likely to increase awareness of low-value treatments among the practitioners providing those procedures. Further, if Choosing Wisely is to have a meaningful impact on reducing healthcare waste, ensuring society recommendations target income-generating treatments provided by their own members is important.

Societies may be naturally cautious about publishing recommendations that could reduce the income of their members and may consequently publish recommendations (unintentionally or intentionally) that apply to non-members or use qualified wording (‘not complete or absolute’). For example, societies primarily involved in providing surgical procedures could target non-surgical treatments provided by non-members or focus on recommendations targeting low-value diagnostic tests. Although this practice could still raise awareness of low-value tests and treatments, there is a missed opportunity to raise awareness of low-value and costly procedures provided by members of that society. Recommendations that are qualified (and therefore open to interpretation) might also limit the impact Choosing Wisely has on reducing low-value care. The wording of practice recommendations is an important factor for increasing adoption [8]. Guidelines advocating that recommendations are actionable (e.g. “don’t”), avoid ambiguity, and are clear enough as a stand-alone resource [9–11]. Using qualified phrases like “when possible”, “don’t routinely” or “unless necessary” may be used to capture clinical complexity but they also give clinicians some latitude to choose an interpretation that avoids practice change.

The primary aim of this study is to determine the proportion of Choosing Wisely recommendations targeting income-generating treatments, and whether society recommendations on income-generating treatments are more likely to target members or non-members. The secondary aim is to determine the prevalence of qualified statements, and whether qualified statements are more likely to appear in recommendations targeting income-generating or non-income-generating treatments that apply to members. We hypothesise that society recommendations on income-generating treatments are less likely to target members compared to non-members, and that qualified statements are more likely to appear in recommendations targeting income-generating treatments (compared to non-income-generating treatments) that apply to members.

## Methods

We performed a content analysis of all published Choosing Wisely recommendations; extracting data from Choosing Wisely websites and The DIANA (Dissemination of Initiatives to ANalyse Appropriateness) in Healthcare website (http://dianasalud.com/index.php) until June 2018. Duplicates were excluded and all recommendations (including corresponding data on the professional society, year, and country) were copied into a spreadsheet. We used the following rules to code each recommendation.

### Characteristics of recommendations

#### Diagnostic test or treatment

Recommendations targeting procedures that are performed to identify or confirm the presence of disease or injury were coded as *‘diagnostic tests’*. Recommendations targeting interventions provided to individuals with disease or injury (excluding diagnostic tests) were coded as *‘treatments’*. Recommendations targeting diagnostic tests and treatments were coded as *‘both’*, while recommendations that did not involve the patient were coded as *‘neither’* (e.g. *“Don’t replace hand hygiene with the use of non-sterile disposable gloves”*).

#### For or against a procedure

Recommendations were coded as *‘don’t’* if they advised against a procedure and *‘do’* if they recommended a procedure. Most of the time the distinction between a *‘do’* and *‘don’t’* was clear. For example, *“Don’t use glucosamine and chondroitin to treat patients with symptomatic osteoarthritis of the knee”* compared to *“Offer PSA screening for detecting prostate cancer only after engaging in shared decision making.”* However, some *‘do’* recommendations were less clear by starting with the word ‘don’t’. For example, *“Don’t delay basic level palliative care for women with advanced or relapsed gynaecologic cancer, and when appropriate, refer to specialty level palliative medicine”.* We coded these recommendations as *‘do’*. Recommendations that were coded as ‘*don’t*’ but provided an alternative to de-implementation were noted. For example, “*Do not use percutaneous feeding tubes in patients with advanced dementia; instead use oral assisted feeding.”*

#### Qualified statements

Recommendations that contained ‘qualified statements’ (statements that were ‘not complete or absolute’) typically used words or phrases like, ‘consider avoiding’, ‘don’t routinely’, ‘don’t overuse’, ‘don’t rely on’, ‘don’t use X unless necessary’, ‘don’t use X without an appropriate clinical indication’. For example, *“Don’t routinely transfuse patients with sickle cell disease (SCD) for chronic anemia or uncomplicated pain crisis without an appropriate clinical indication”*. Examples of qualified statements are outlined in Additional file 1. Recommendations that we considered clear and absolute were coded as *‘unqualified’*. For example, *“Don’t transfuse red blood cells in hemodynamically stable, non-bleeding ICU patients with a hemoglobin concentration greater than 7 g/dL.”*

#### Income-generating treatments

Treatments that attract a fee for service and are performed outside of a routine clinical encounter (i.e. a scheduled procedure) were coded as ‘income-generating’ for the clinician performing the treatment (e.g. surgery, insertion of an instrument or device for a therapeutic purpose, and radiotherapy). The full list of income-generating treatments found among Choosing Wisely recommendations is reported in Additional file 1. ‘Non-income-generating’ treatments included, but were not limited to, medication, electrotherapy, injection therapies (excluding vitreal injections), blood transfusions, inserting a catheter, and administering supplemental oxygen. These treatments could not be considered ‘income-generating’ by the above definition (i.e. they could be provided within a routine clinical encounter).

#### Recommendations that apply to members

Recommendations that targeted treatments provided or performed by members of the society generating the recommendation were considered to ‘apply to members’. We did not code recommendations for diagnostic tests because radiologists and pathologists provide or perform the majority of these tests in most healthcare settings (excluding the use of diagnostic tests by, for example, emergency physicians, general physicians and cardiologists). In other words, most recommendations targeting diagnostic tests would be coded as ‘non-income-generating’ and ‘apply to non-members’ if not published by a diagnostic society. We excluded *‘do’* recommendations, recommendations from societies not linked with the delivery of health services (e.g. *The International Society of Doctors for the Environment*), and recommendations from groups of societies or societies that had members across health disciplines (e.g. nursing, surgery, medicine).

#### Rater panel

The panel was comprised of an orthopaedic surgeon, a senior resident in orthopaedic surgery, and two physiotherapists; all of whom were clinician researchers (two were professors; three had PhDs). Collectively the panel had decades of clinical experience in a range of healthcare settings in Australia and abroad.

### Data analysis

All four researchers independently coded the first 150 recommendations to assess agreement and refine the coding rules. Once the researchers agreed upon the final coding rules, two researchers, drawn from the panel of four, independently coded all the recommendations, resolving all disagreements by discussion or consultation with a third researcher where necessary. Although two researchers independently coded the recommendations and all disagreements were resolved, we were interested in testing the reliability of the coding framework. Kappa statistics (95% confidence intervals (CI)) and percent exact agreement were calculated to assess agreement between reviewers for coding the characteristics of all recommendations. Kappa (k) statistics were interpreted as follows: < 0.00 = “poor”, 0.00–0.20 = “slight”, 0.21–0.40 = “fair”, 0.41–0.60 = “moderate”, 0.61–0.80 = “substantial”, ≥0.81 = “almost perfect” [12].

Descriptive statistics (counts and percentages) were used to report the coded data, as well as the number of recommendations, and the professional societies that published Choosing Wisely lists (including the country). A Chi-squared test evaluated whether society recommendations on income-generating treatments are more likely to target members or non-members; and whether qualified statements were more likely to appear in recommendations targeting income-generating or non-income-generating treatments that apply to members. We then stratified our main analyses by society (categorised as medical, surgical, diagnostic, allied health – including nursing, pharmacy and dentistry – and other). All analyses were performed in STATA statistical software (version 13.1).

## Results

We found 1293 Choosing Wisely recommendations across the United States (*n* = 535, 41.4%), Canada (*n* = 309, 23.9%), Italy (*n* = 174, 13.5%), Australia (*n* = 172, 13.3%), the United Kingdom (*n* = 56, 4.3%), Netherlands (*n* = 30, 2.3%), New Zealand (*n* = 12, 0.9%; 117 duplicate recommendations were excluded), and Japan (*n* = 5, 0.4%) (Table 1). Choosing Wisely campaigns were also found (or mentioned) in Austria, Brazil, Denmark, France, Israel, Portugal, South Korea, Switzerland, and Wales but no recommendations were readily available. Germany published 10 recommendations only intended to guide the development of future recommendations, and these were therefore not included in the analysis. Of the included recommendations, 646 (50.0%) were from medical societies, 243 (18.8%) from surgical societies, 181 (14.0%) from diagnostic societies (e.g. radiology, pathology), 137 (10.6%) from allied health societies (including nursing, pharmacy and dentistry), and 86 (6.7%) from other societies (Table 1). A detailed breakdown of the societies across countries is in Additional file 2.

**Table 1:** Characteristics of Choosing Wisely recommendations

Country	n	%
United States	535	41.4
Canada	309	23.9
Italy	174	13.5
Australia	172	13.3
United Kingdom	56	4.3
Netherlands	30	2.3
New Zealand^a^	12	0.9
Japan	5	0.4
Society	n	%
Medical	646	50.0
Surgical	243	18.8
Diagnostic	181	14.0
Allied health^b^	137	10.6
Other	86	6.7
Diagnostic test or treatment	n	%
Test	624	48.3
Treatment	628	48.6
Both	24	1.8
Neither	17	1.3
For or against a procedure	n	%
Do	83	6.4
Don’t	1210	93.6
Alternative provided (if *‘Don’t’*)	55	4.3
Wording	n	%
Qualified	542	41.9
Unqualified	751	58.1
Treatment recommendations (*n* = 552)^c^	n	%
Apply to members^d^	505	91.5
Apply to non-members^d^	47	8.5
Income-generating^d^	98	17.8
Non-income-generating^d^	454	82.3

*n* Number of recommendations %: percentage of all recommendations (unless otherwise specified); ^a^number of unique recommendations mentioned in Choosing Wisely New Zealand; ^b^including nursing, dentistry and pharmacy

^c^excluding treatment recommendations promoting a procedure (*n* = 58), from multidisciplinary societies (*n* = 33), from societies not directly linked to the delivery of health services (*n* = 9); ^d^percentage of 552 treatment recommendations

### Characteristics of choosing wisely recommendations

There were 624 (48.3%) recommendations concerning diagnostic tests, 628 (48.6%) concerning treatments, 24 (1.8%) concerning both, and 17 (1.3%) that did not involve patient care so could not be classified as either a diagnostic test or treatment. There were 1210 (93.6%) recommendations that advised against a procedure and 83 (6.4%) that recommended a procedure. Of the 1210 recommendations that advised against a procedure, only 55 (4.5%) provided an alternative test or treatment. There were 98 (17.8%) recommendations targeting income-generating treatments and 454 (82.3%) recommendations targeting non-income-generating treatments (Table 1). The coding of all recommendations can be found in Additional file 3.

Agreement between reviewers for coding recommendations was ‘almost perfect’ for diagnostic tests or treatments (k = 0.94, 95% CI: 0.92 to 0.95), ‘substantial’ for qualified statements (k = 0.71, 95% CI: 0.67 to 0.75) and income-generating treatments (k = 0.79, 95% CI: 0.71 to 0.85), and ‘moderate’ for recommendations for or against a procedure (k = 0.48, 95% CI: 0.37 to 0.57) and recommendations that apply to members (k = 0.42, 95% CI: 0.29 to 0.55) (Table 2). Percent exact agreement was high across all coding domains (range: 86 to 97%).

**Table 2:** Kappa and percent exact agreement between reviews for coding characteristics of recommendations

Characteristic of recommendations	*n*	Agreement	k	95% CI
Diagnostic test or treatment	1293	97%	0.94	0.92–0.95
For or against a procedure	1293	94%	0.48	0.37–0.57
Qualified statements	1293	86%	0.71	0.67–0.75
Income-generating treatments	552	94%	0.79	0.71–0.85
Apply to members	552	91%	0.42	0.29–0.55

*n* Number of recommendations, *k* Kappa coefficient, *CI* Confidence interval

### Income-generating treatments that apply to members

**Fig. 1:**
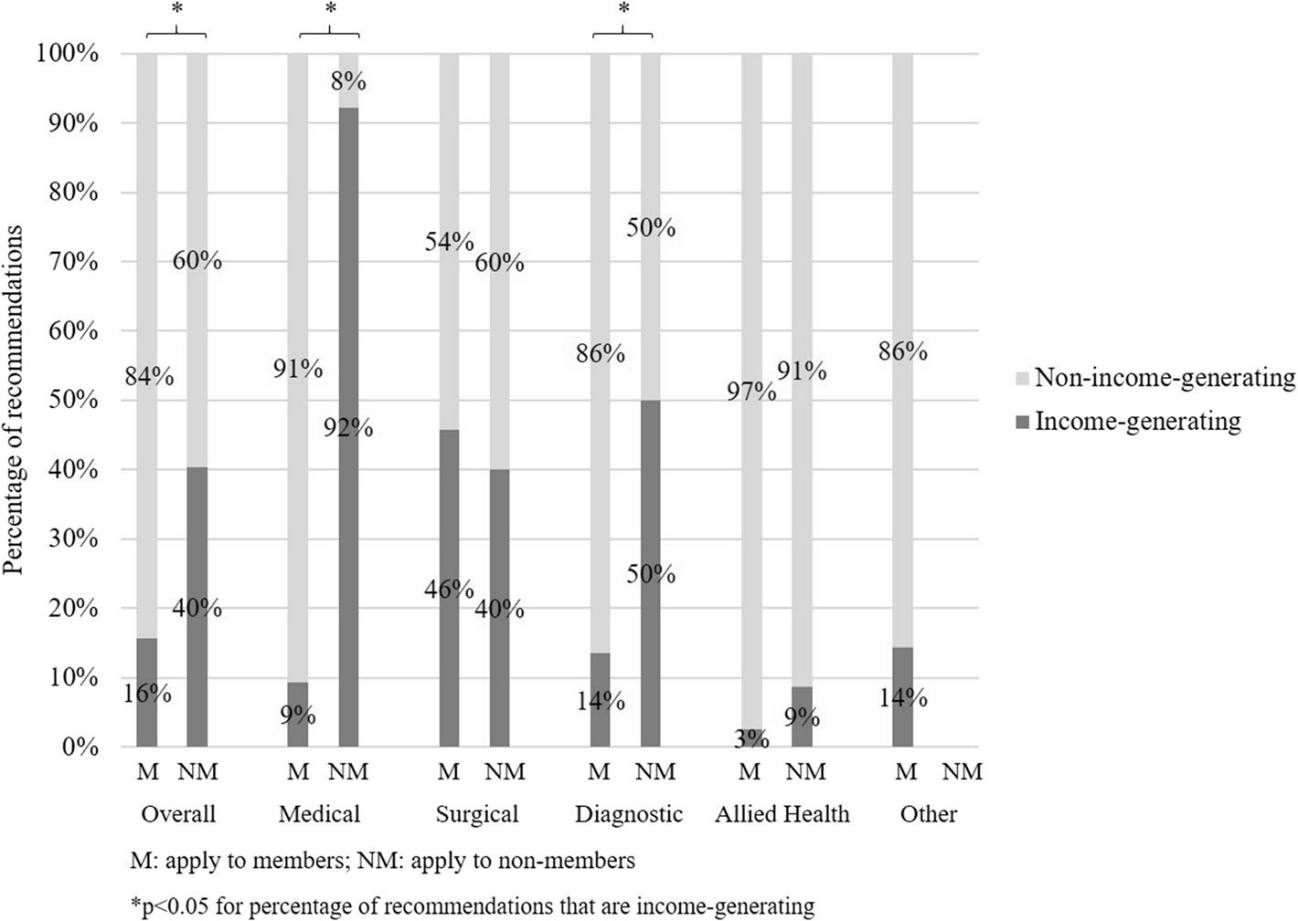
Target of treatment recommendations across societies

There were 98 (17.8%) treatment recommendations targeting income-generating treatments, and recommendations regarding income-generating treatments were less likely to target members compared to non-members (15.6% vs. 40.4%, *p* < 0.001) (Table 3) (Fig. 1). Recommendations targeting income-generating treatments were most common across surgical societies (45.5%) and least common across medical (12.9%) and allied health societies (4.0%). Moreover, recommendations regarding income-generating treatments were only less likely to target members compared to non-members across medical (9.3% vs. 92.3%, *p* < 0.001) and diagnostic societies (13.6% vs. 50.0%, *p* = 0.03) (Fig. 1).

**Table 3:** Treatment recommendations (*n* = 552)

	Income-generating	Non-income-generating			
	*n*	%	*n*	%	
Apply to members^a^	79	15.6	426	84.4	*p* < 0.001, Chi^2^ = 18.1
Apply to non-members^b^	19	40.4	28	59.6	

*n* Number of recommendations; ^a^percentage of treatment recommendations that apply to members; ^b^percentage of treatment recommendations that apply to non-members

### Qualified wording

Five-hundred and forty-two (41.9%) recommendations were qualified and 751 (58.1%) were unqualified (Table 1), with medical (46.6%) and surgical societies (42.0%) displaying the highest proportion of ‘qualified’ recommendations (Additional file 4). A similar proportion of recommendations targeting income-generating and non-income-generating treatments that apply to members contained qualified statements (49.4% vs. 42.0%, *p* = 0.23). (Table 4). There was no difference in the proportion of qualified statements among all income-generating and non-income-generating treatment recommendations (45.9% vs. 41.4%, *p* = 0.41), and among treatment recommendations targeting members and non-members (43.2% vs. 31.9%, *p* = 0.16) (Additional file 5).

**Table 4:** Wording of treatment recommendations that apply to members (*n* = 426)

	Qualified		Unqualified		
	*n*	%	*n*	%	
Income-generating^a^	39	49.4	40	50.6	*p* = 0.23, Chi^2^ = 1.5
Non-income-generating^b^	179	42.0	247	58.0	

n: number of recommendations; ^a^percentage of income-generating treatments; ^b^percentage of non-income generating treatments

## Discussion

Choosing Wisely recommendations are framed in ways that lessen potential impact on the members of the society making the recommendation. Only 20% of treatment recommendations refer to income-generating treatments, and these are less likely to target members compared to non-members. Although nearly half of Choosing Wisely recommendations are qualified, qualified statements were not more likely to appear in recommendations targeting income-generating treatments that apply to members. Societies need to generate recommendations that specifically target the practice of members, and reduce, if not eliminate, qualified recommendations from Choosing Wisely.

### Strengths and weakness of the study

This study has numerous strengths, including being the first to evaluate the content of all Choosing Wisely recommendations. We searched both Choosing Wisely websites and The DIANA in Healthcare website to ensure a systematic evaluation of all available recommendations. The method used to code the data was robust as four researchers independently rated 150 recommendations and discussed disagreements to pilot the checklist. The research team had decades of clinical experience in a range of healthcare settings in Australia and abroad. This experience was sufficient to develop a reliable coding tool; percent exact agreement ranged from 86 to 97% across all coding domains. Further, any limitations of the coding tool are unlikely to influence the results since two researchers independently rated all 1293 recommendations and resolved any disagreements by discussion or consultation with one of the other two researchers. The primary limitation is that differences in reimbursement systems between countries might influence what treatments generate income for clinicians. Nevertheless, we are confident the treatments classified as ‘income-generating’ (Additional file 1) would remain in this category across most healthcare systems.

### Meaning of the study

Choosing Wisely aims to reduce waste in healthcare. However, pursuit of this aim is arguably compromised when society recommendations on income-generating treatments are less often targeted towards members compared to non-members. In addition, almost half of Choosing Wisely recommendations target low-value diagnostic tests, with only 20% of test recommendations coming from diagnostic societies. Increasing awareness of low-value testing is important, but when societies mainly look for waste in fields other than their own, their recommendations are likely to have less impact [6, 7]. To illustrate these points, 23 societies have published recommendations about imaging for low back pain, and five have published recommendations about imaging for acute ankle sprains. In contrast, of the eight societies that include orthopaedic surgeons (*n* = 48 recommendations), there are only 9 recommendations about low-value surgery (e.g. knee arthroscopy, spinal fusion and subacromial decompression) [13–15].

Choosing Wisely recommendations are often qualified, using phrases like ‘don’t routinely’, ‘don’t overuse’ or ‘unless necessary’. Although qualified statements may be necessary for some areas of practice, the counter argument is that they give clinicians latitude to adopt an interpretation of the recommendation that best suits their practice and make it difficult to appropriately measure adherence [8, 16, 17]. With this in mind, it might not be surprising that 1 year after the launch of the campaign there was little-to-no change in the de-implementation of a number of low-value practices in the United States (e.g. imaging for low back pain and human papillomavirus testing in young women) [18]. We acknowledge that this evaluation was limited to a small number of recommendations and that increased awareness of Choosing Wisely since it was launched in 2012 might have increased the impact it has had on reducing low-value care [1]. Nevertheless, if Choosing Wisely is to maximise its impact on improving patient care and reducing the enormous amount of healthcare waste worldwide [19–21], recommendations may need to be unqualified.

### Unanswered questions and future research

A few studies suggest some societies are averse to targeting income-generating treatments provided by their members [7, 22, 23], and instead “pick the low hanging fruit that was so low it was lying on the ground” (Vikas Saini – cardiologist and president of the Lown Institute) [7]. Although this behaviour is understandable, research exploring the drivers of this behaviour is essential if Choosing Wisely hopes to realise its goal of reducing low-value care and healthcare waste. A recent editorial [24] on *‘Why do surgeons continue to perform unnecessary surgery?’* provided some insight on these barriers from a surgeon’s perspective. The authors suggest that surgeons continue to provide low-value surgery because of incentives – either from income, status, or both – and because they are trained to do surgery. Further, selective anecdotes of successful cases and years of education and training might foster a strong attachment to surgery, making de-implementation even more difficult [25]. With this in mind, surgical societies might be the most averse to targeting income-generating treatments provided by members. However, we found this was not the case. The proportion of recommendations targeting income-generating treatments provided by members was highest across surgical societies (45.5%), and much lower across medical (12.9%) and diagnostic societies (18.0%). In addition, society recommendations on income-generating treatments were significantly less likely to target members compared to non-members across medical and diagnostic societies, but not across surgical societies. These findings are likely explained by many recommendations from medical societies targeting surgical procedures and many recommendations from diagnostic societies targeting treatments. Medical and diagnostic societies should therefore consider how their Choosing Wisely lists could better target unnecessary and high-cost care provided by their members.

Investigating strategies to increase adoption of Choosing Wisely recommendations should be a research priority. Compared to more intensive implementation strategies such as audit and feedback or educational outreach, refining the wording of Choosing Wisely recommendations is simple and cheap, making it a logical first step towards increasing adoption among clinicians. Re-wording recommendations so they are unqualified will send a clear message to clinicians and is necessary to ensure de-implementation is measurable [8]. Further, there is underuse of recommendations that provide an alternative to de-implementation and including ‘do’ and ‘don’t’ statements within a recommendation could increase clinicians’ willingness to replace low-value care with the suggested high-value alternative. The evaluative linguistic framework developed by Buchbinder and colleagues also shows promise for judging and enhancing the quality of medical information (e.g. drug leaflets) [26, 27] and could be adapted to evaluate the quality of Choosing Wisely recommendations. For example, the tool judges the clarity of what ‘action’ must be taken, who is responsible for the ‘action’, and the importance or urgency of the ‘action’. Ensuring Choosing Wisely recommendations clearly address these factors could be vital for increasing clinicians’ willingness to follow them. The next step for Choosing Wisely should be research investigating how the wording of Choosing Wisely recommendations influences adoption, as this will have important implications for refining existing recommendations and developing new ones.

## Conclusion

Our analysis found that Choosing Wisely recommendations made by societies are often framed to minimise impact on their members. Only 20% of recommendations target income-generating treatments, and of these recommendations most target non-members. Nearly half of Choosing Wisely recommendations are qualified. Increasing the proportion of Choosing Wisely recommendations that are unqualified and target member clinicians responsible for de-implementation of low-value treatments should be a priority.

## Supplementary information

**Additional file 1.** Examples of qualified statements and income-generating treatments.

**Additional file 2.** Choosing Wisely recommendations across individual societies and country.

**Additional file 3.** Coding of all recommendations.

**Additional file 4.** Wording of Choosing Wisely recommendations across societies. n: number of recommendations; %: percentage of all recommendations within each society; *including nursing, dentistry and pharmacy.

**Additional file 5.** Wording of treatment recommendations (*n* = 552). n: number of recommendations; *: percentage of income-generating treatments; †: percentage of non-income generating treatments; ‡: percentage of treatment recommendations provided by members; §: percentage of treatment recommendations provided by non-members.

## Data Availability

All data generated or analysed during this study are included in this published article (see Additional file 3).

## Abbreviations

ABIM: American Board of Internal Medicine
CI: Confidence interval
DIANA: Dissemination of Initiatives to ANalyse Appropriateness
k: Kappa
