# Supplementary material for: Do choosing wisely recommendations about low-value care target income-generating treatments provided by members? A content analysis of 1293 recommendations
Source: BMC Health Serv Res. 2019 Nov 11;19:707. doi: 10.1186/s12913-019-4576-1 (PMC6844045; doi:10.1186/s12913-019-4576-1)
Supplement: Supplementary file 1 — Additional file 1. Examples of qualified statements and income-generating treatments. [file 12913_2019_4576_MOESM1_ESM.docx]

| Additional file 1. Examples of qualified statements and income-generating treatments | |
| --- | --- |
| **QUALIFIED STATEMENTS** | |
| **Examples of words or phrases that are qualified** | Avoid… |
|  | Consider… |
|  | Don’t overuse… |
|  | Don’t rely on… |
|  | Don’t rely solely on… |
|  | Don’t routinely… |
|  | Don’t treat X indiscriminately |
|  | Don’t use X as a first choice |
|  | Don’t use X for long periods |
|  | Don’t use X regularly |
|  | Don’t use X without regular attempts at… |
|  | Don’t use X except in rare circumstances |
|  | Don’t use X unless absolutely necessary |
|  | Don’t use X unless clinically indicated |
|  | Don’t use X unless clinically necessary |
|  | Don’t use X unless there is a clinical indication |
|  | Don’t use X unless there is a reasonable expectation of benefit |
|  | Don’t use X unless there is a valid clinical indication |
|  | Don’t use X where possible |
|  | Don’t usually… |
|  | In general there is no indication to… |
|  | Reconsider the use of… |
|  | Reduce use of… |
|  | Restrict the use of… |
|  | X is not necessary |
|  | X should be avoided |
|  | X should not be standard practice |
| **Examples where the clinical indication is ambiguous** | *“Electronic monitoring of a baby’s heart should not be offered routinely during labour unless the mother is at a higher risk of complications than normal.”*  Royal College of Obstetricians & Gynaecologists (United Kingdom) |
|  | *“Don't order sinus computed tomography (CT) or indiscriminately prescribe antibiotics for uncomplicated acute rhinosinusitis.”*  American Academy of Allergy, Asthma & Immunology (United States) |
|  | *“Thyroid nodules patients should not be treated with L-thyroxine except in selected cases*”  The Italian Association of Medical Endocrinologists (Italy) |
|  | *“Don't place or maintain a urinary catheter in a patient unless there is a specific indication to do so.”*  American Academy of Nursing (United States) |
|  | *“Don't perform preoperative medical tests for eye surgery unless there are specific medical indications.”*  American Academy of Ophthalmology (United States) |
|  | *“Don't routinely remove the gallbladder unless clinically indicated.”*  American Society for Metabolic and Bariatric Surgery (United States) |
|  | *“Do not order herpes serology tests unless there is a clear clinical indication”* Australiasian Chapter of Sexual Health Medicine (Australia) |
|  | *“Don't perform stress cardiac imaging or advanced non-invasive imaging in the initial evaluation of patients without cardiac symptoms unless high-risk markers are present.”*  American College of Cardiology (United States) |
|  | *“Don't routinely measure Vitamin D levels in low risk adults.”*  Nurse Practitioner: Nurse Practitioner Association of Canada (Canada) |
|  | *“Don't perform pelvic ultrasound in average risk women to screen for ovarian cancer.”*  American College of Obstetricians and Gynecologists (United States) |
| **Examples that provide weak exceptions** | *“After treatment for cancer, the use of routine scanning should only be used where this is beneficial to the patient.”*  Royal College of Radiologists (United Kingdom) |
|  | *“Don't prescribe antipsychotic medications to patients for any indication without appropriate initial evaluation and appropriate ongoing monitoring.”*  American Psychiatric Association (United States) |
|  | *“Don't prescribe antipsychotic medications for behavioral and psychological symptoms of dementia (BPSD) in individuals with dementia without an assessment for an underlying cause of the behavior.”*  AMDA – The Society for Post-Acute and Long-Term Care Medicine (United States) |
|  | *“Don't overuse non-beta lactam antibiotics in patients with a history of penicillin allergy, without an appropriate evaluation.”*  American Academy of Allergy, Asthma & Immunology (United States) |
|  | *“Don't prescribe opioid pain medication in pregnancy without discussing and fully weighing the risks to the woman and her fetus.”*  American Academy of Nursing (United States) |
|  | *“Don't perform screening panels for food allergies without previous consideration of medical history.”*  American Academy of Pediatrics (United States) |
|  | *“Don't prescribe bed rest for acute localized back pain without completing an evaluation.”*  American Academy of Physical Medicine and Rehabilitation (United States) |
|  | *“Don't order an imaging study for back pain without performing a thorough physical examination.”*  American Academy of Physical Medicine and Rehabilitation (United States) |
|  | *“Don't do nerve conduction studies without also doing a needle EMG for testing for radiculopathy, a pinched nerve in the neck or back.”*  American Association of Neuromuscular & Electrodiagnostic Medicine (United States) |
|  | *“Don't initiate management of low-risk prostate cancer without discussing active surveillance.”*  American Society for Radiation Oncology (United States) |
| **Examples with no instruction** | *“Unless a patient is at risk of prostate cancer because of race or family history, PSA-based screening does not lead to a longer life.”*  Royal College of Pathologists (United Kingdom) |
|  | *“In cases of a minor head injury, imaging is not likely to be useful”*  Royal College of Radiologists (United Kingdom) |
| **INCOME-GENERATING TREATMENTS** | |
| **List of treatments** | Ablation |
|  | Anterior cruciate ligament reconstruction |
|  | Arthroscopy |
|  | Axillary lymph node dissection |
|  | Bariatric surgical |
|  | Caesarean section |
|  | Carpal tunnel release |
|  | Double mastectomy |
|  | Draining fluid-filled breast cysts |
|  | Episiotomy |
|  | Excising areas of psuedoangiomatous stromal hyperplasia |
|  | Excising fibroadenomas |
|  | Fasciotomy |
|  | Hip or knee arthroplasty |
|  | Hysterectomy |
|  | Implanting an implantable cardioverter defibrillator or pacemaker |
|  | Inducing labour |
|  | Inserting a cervical cerclage |
|  | Inserting an inferior vena cava filter |
|  | Inserting ear tubes |
|  | Inserting percutaneous feeding tubes |
|  | Inserting punctual plugs |
|  | Intravitreal injections |
|  | Lumbar fusion surgery |
|  | Mohs micrographic surgery |
|  | Percutaneous coronary interventions or bypass surgery |
|  | Radiotherapy |
|  | Rectocele repairs |
|  | Removing a breast lump |
|  | Removing a gallbladder |
|  | Removing cancer |
|  | Removing dental amalgams |
|  | Removing or applying braces, occlusal equilibration and restorations |
|  | Removing ovaries at hysterectomy |
|  | Removing synthetic vaginal mesh |
|  | Repairing a hernia |
|  | Repairing an abdominal aortic aneurysm |
|  | Retinal laser |
|  | Robotic assisted laparoscopic surgery |
|  | Subacromial decompression |
|  | Surgery for a narrowed carotid artery |
|  | Surgery for CIN 1 lesion |
|  | Surgery for plantar fasciitis |
