# Supplementary material for: Do choosing wisely recommendations about low-value care target income-generating treatments provided by members? A content analysis of 1293 recommendations
Source: BMC Health Serv Res. 2019 Nov 11;19:707. doi: 10.1186/s12913-019-4576-1 (PMC6844045; doi:10.1186/s12913-019-4576-1)
Supplement: Supplementary file 2 — Additional file 2. Choosing Wisely recommendations across individual societies and country. [file 12913_2019_4576_MOESM2_ESM.docx]

| Additional file 2. Choosing Wisely recommendations across individual societies and country | | | | | | | | | |
| --- | --- | --- | --- | --- | --- | --- | --- | --- | --- |
|  | US | Canada | Italy | Australia | UK | Netherlands | Japan | NZ | Total |
| **MEDICAL** | 255 | 169 | 65 | 96 | 36 | 15 | 5 | 5 | 646 |
| Pediatrics | 10 | 5 | 5 | 5 | 4 | 5 |  |  | 34 |
| Emergency physicians | 10 | 10 |  | 6 | 5 |  |  |  | 31 |
| Internal medicine | 5 | 6 | 10 | 5 |  | 5 |  |  | 31 |
| General practice |  |  | 5 | 10 | 10 |  | 5 |  | 30 |
| Radiation Oncology | 10 | 10 | 5 | 5 |  |  |  |  | 30 |
| Family physicians | 15 | 13 |  |  |  |  |  |  | 28 |
| Dermatology | 10 |  |  | 5 |  | 5 |  | 5 | 25 |
| Gastroenterology | 5 | 10 | 5 | 5 |  |  |  |  | 25 |
| Psychiatry | 5 | 13 |  |  | 4 |  |  |  | 22 |
| Endocrine | 5 | 5 | 5 | 5 |  |  |  |  | 20 |
| Geriatrics | 10 | 5 |  | 5 |  |  |  |  | 20 |
| Infectious disease | 5 | 10 |  | 5 |  |  |  |  | 20 |
| Palliative | 5 | 5 | 5 | 5 |  |  |  |  | 20 |
| Physical medicine and rehabilitation | 5 | 6 |  | 5 |  |  |  |  | 16 |
| Blood transfusion |  | 15 |  |  |  |  |  |  | 15 |
| Cardiology | 5 | 5 | 5 |  |  |  |  |  | 15 |
| Clinical oncology | 10 |  | 5 |  |  |  |  |  | 15 |
| Nephrology | 5 | 5 | 5 |  |  |  |  |  | 15 |
| Occupational and Environmental medicine | 5 | 5 |  | 5 |  |  |  |  | 15 |
| Opthamologists | 5 |  |  | 5 | 5 |  |  |  | 15 |
| Rheumatology | 5 | 5 |  | 5 |  |  |  |  | 15 |
| Adult and pediatric Hospital medicine | 10 |  |  |  |  |  |  |  | 10 |
| AMDA – The Society for Post-Acute and Long-Term Care Medicine | 10 |  |  |  |  |  |  |  | 10 |
| Critical care | 5 | 5 |  |  |  |  |  |  | 10 |
| Liver diseases | 5 | 5 |  |  |  |  |  |  | 10 |
| Maternal-fetal Medicine | 10 |  |  |  |  |  |  |  | 10 |
| Pediatrics - Endocrinology | 5 |  |  | 5 |  |  |  |  | 10 |
| Preventive Medicine | 5 |  | 5 |  |  |  |  |  | 10 |
| Reproductive Medicine | 10 |  |  |  |  |  |  |  | 10 |
| Sports Medicine | 5 | 5 |  |  |  |  |  |  | 10 |
| Intensive care |  |  |  | 5 | 4 |  |  |  | 9 |
| Sexual health medicine |  |  |  | 5 | 4 |  |  |  | 9 |
| Long-term care |  | 6 |  |  |  |  |  |  | 6 |
| Respiratory medicine: Thoracic society | | 6 |  |  |  |  |  |  | 6 |
| Blood banks | 5 |  |  |  |  |  |  |  | 5 |
| Cardiovascular Angiography and Interventions | 5 |  |  |  |  |  |  |  | 5 |
| Chest physicians & Thoracic Society | 5 |  |  |  |  |  |  |  | 5 |
| Gynecologic Oncology | 5 |  |  |  |  |  |  |  | 5 |
| HIV Medicine | 5 |  |  |  |  |  |  |  | 5 |
| Hospital medicine |  | 5 |  |  |  |  |  |  | 5 |
| Pediatric allergy |  |  | 5 |  |  |  |  |  | 5 |
| Pediatric Ophthalmology and Strabismus | 5 |  |  |  |  |  |  |  | 5 |
| Pediatric Rheumatology | 5 |  |  |  |  |  |  |  | 5 |
| Pediatrics - Perinatal | 5 |  |  |  |  |  |  |  | 5 |
| Physicians | 5 |  |  |  |  |  |  |  | 5 |
| Sleep medicine | 5 |  |  |  |  |  |  |  | 5 |
| Pediatrics infectious diseases |  | 4 |  |  |  |  |  |  | 4 |
| Vascular medicine | 5 |  |  |  |  |  |  |  |  |
|  |  |  |  |  |  |  |  |  |  |
|  | US | Canada | Italy | Australia | UK | Netherlands | Japan | NZ | Total |
| **SURGICAL** | 110 | 68 | 15 | 25 | 10 | 15 |  |  | 243 |
| Obstetricians and Gynecologists | 10 | 10 | 5 | 5 | 5 |  |  |  | 35 |
| Neurology/neuro surgery | 10 |  | 10 | 5 |  | 5 |  |  | 30 |
| Orthopaedic surgeons | 10 | 10 |  |  |  | 5 |  |  | 25 |
| Urological | 15 | 5 |  |  |  | 5 |  |  | 25 |
| Otolaryngology—Head & Neck Surgery | 10 | 8 |  | 5 |  |  |  |  | 23 |
| Surgeons | 5 | 6 |  | 5 | 5 |  |  |  | 21 |
| Anesthesiologists | 5 | 5 |  | 5 |  |  |  |  | 15 |
| Spine society | 5 | 8 |  |  |  |  |  |  | 13 |
| Pediatrics surgery |  | 11 |  |  |  |  |  |  | 11 |
| Breast surgeons | 10 |  |  |  |  |  |  |  | 10 |
| Surgical oncology | 10 |  |  |  |  |  |  |  | 10 |
| Vascular surgery | 5 | 5 |  |  |  |  |  |  | 10 |
| Metabolic and Bariatric Surgery | 5 |  |  |  |  |  |  |  | 5 |
| Thoracic surgeons | 5 |  |  |  |  |  |  |  | 5 |
| Urogynecologic | 5 |  |  |  |  |  |  |  | 5 |
|  |  |  |  |  |  |  |  |  |  |
|  | US | Canada | Italy | Australia | UK | Netherlands | Japan | NZ | Total |
| **DIAGNOSTIC** | 105 | 20 | 20 | 26 | 10 |  |  |  | 181 |
| Pathology | 20 | 5 |  | 5 | 5 |  |  |  | 35 |
| Radiology | 10 | 5 | 5 | 6 | 5 |  |  |  | 31 |
| Asthma, allergy, immunology | 10 |  | 10 | 5 |  |  |  |  | 25 |
| Hematology | 10 | 5 |  | 5 |  |  |  |  | 20 |
| Nuclear Medicine and Molecular Imaging | 5 | 5 | 5 |  |  |  |  |  | 15 |
| Toxicology | 10 |  |  | 5 |  |  |  |  | 15 |
| Neuromuscular & Electrodiagnostic Medicine | 10 |  |  |  |  |  |  |  | 10 |
| Cardiovascular Computed Tomography | 5 |  |  |  |  |  |  |  | 5 |
| Cardiovascular Magnetic Resonance | 5 |  |  |  |  |  |  |  | 5 |
| Colposcopy and Cervical Pathology | 5 |  |  |  |  |  |  |  | 5 |
| Echocardiography | 5 |  |  |  |  |  |  |  | 5 |
| Heart Rhythm | 5 |  |  |  |  |  |  |  | 5 |
| Nuclear Cardiology | 5 |  |  |  |  |  |  |  | 5 |
|  |  |  |  |  |  |  |  |  |  |
|  | US | Canada | Italy | Australia | UK | Netherlands | Japan | NZ | Total |
| **ALLIED HEALTH** | 45 | 31 | 45 | 16 |  |  |  |  | 137 |
| Nursing | 20 | 25 | 30 | 5 |  |  |  |  | 80 |
| Pharmacy | 5 | 6 | 5 | 5 |  |  |  |  | 21 |
| Physiotherapy | 5 |  | 5 | 6 |  |  |  |  | 16 |
| Chiropractors | 5 |  |  |  |  |  |  |  | 5 |
| Dentists | 5 |  |  |  |  |  |  |  | 5 |
| Dietetics and Nutrition |  |  | 5 |  |  |  |  |  | 5 |
| Podiatry | 5 |  |  |  |  |  |  |  | 5 |
|  |  |  |  |  |  |  |  |  |  |
|  | US | Canada | Italy | Australia | UK | Netherlands | Japan | NZ | Total |
| **OTHER** | 20 | 20 | 30 | 9 |  |  |  | 7 | 86 |
| Education |  | 11 | 5 |  |  |  |  | 7 | 23 |
| Genetics | 5 | 5 | 5 | 5 |  |  |  |  | 20 |
| Headache/pain | 10 | 4 |  | 4 |  |  |  |  | 18 |
| Forensic, biochemistry, environment |  |  | 15 |  |  |  |  |  | 15 |
| Cardiovascular prevention epidemiology |  |  | 5 |  |  |  |  |  | 5 |
| Healthcare Epidemiology | 5 |  |  |  |  |  |  |  | 5 |
