# Supplementary material for: Do choosing wisely recommendations about low-value care target income-generating treatments provided by members? A content analysis of 1293 recommendations
Source: BMC Health Serv Res. 2019 Nov 11;19:707. doi: 10.1186/s12913-019-4576-1 (PMC6844045; doi:10.1186/s12913-019-4576-1)
Supplement: Supplementary file 4 — Additional file 4. Wording of Choosing Wisely recommendations across societies. n: number of recommendations; %: percentage of all recommendations within each society; *including nursing, dentistry and pharmacy. [file 12913_2019_4576_MOESM4_ESM.docx]

| Additional file 4. Wording of Choosing Wisely recommendations across societies. | | | | | | | | | | | | | | |
| --- | --- | --- | --- | --- | --- | --- | --- | --- | --- | --- | --- | --- | --- | --- |
|  | **Medical** | |  | **Surgical** | |  | **Diagnostic** | |  | **Allied health*** | |  | **Other** | |
| **Wording** | **n** | **%** |  | **n** | **%** |  | **n** | **%** |  | **n** | **%** |  | **n** | **%** |
| **Qualified** | 301 | 46.6 |  | 102 | 42.0 |  | 57 | 31.5 |  | 54 | 39.4 |  | 28 | 32.6 |
| **Unqualified** | 345 | 53.4 |  | 141 | 58.0 |  | 124 | 68.5 |  | 83 | 60.6 |  | 58 | 67.4 |

n: number of recommendations; %: percentage of all recommendations within each society
*including nursing, dentistry and pharmacy
