# Supplementary material for: Do choosing wisely recommendations about low-value care target income-generating treatments provided by members? A content analysis of 1293 recommendations
Source: BMC Health Serv Res. 2019 Nov 11;19:707. doi: 10.1186/s12913-019-4576-1 (PMC6844045; doi:10.1186/s12913-019-4576-1)
Supplement: Supplementary file 5 — Additional file 5. Wording of treatment recommendations (n = 552). n: number of recommendations; *: percentage of income-generating treatments; †: percentage of non-income generating treatments; ‡: percentage of treatment recommendations provided by members; §: percentage of treatment recommendations provided by non-members. [file 12913_2019_4576_MOESM5_ESM.docx]

Additional file 5. Wording of treatment recommendations (n=552)

|  | **Income-generating*** | |  | **Non-income-generating**† | |  |  | **Apply to members**‡ | |  | **Apply to non-members**§ | |  |
| --- | --- | --- | --- | --- | --- | --- | --- | --- | --- | --- | --- | --- | --- |
| **Wording** | **n** | **%** |  | **n** | **%** |  |  | **n** | **%** |  | **n** | **%** |  |
| **Qualified** | 45 | 45.9 |  | 188 | 41.4 | p=0.412,  Chi^2^= 0.7 |  | 218 | 43.2 |  | 15 | 31.9 | p=0.135,  Chi^2^= 2.2 |
| **Unqualified** | 53 | 54.1 |  | 266 | 58.6 |  |  | 287 | 56.8 |  | 32 | 68.1 |  |

n: number of recommendations; *: percentage of income-generating treatments; †: percentage of non-income generating treatments;
‡: percentage of treatment recommendations provided by members; §: percentage of treatment recommendations provided by non-members.
